# Hymenopteran Parasitoids of Hard Ticks in Western Africa and the Russian Far East

**DOI:** 10.3390/microorganisms8121992

**Published:** 2020-12-14

**Authors:** Mapenda Gaye, Nadia Amanzougaghene, Younes Laidoudi, El Hadji Amadou Niang, Zuzana Sekeyová, Maureen Laroche, Jean-Michel Bérenger, Didier Raoult, Mária Kazimírová, Florence Fenollar, Oleg Mediannikov

**Affiliations:** 1 Aix Marseille University, Institut de Recherche pour le Développement (IRD), Assistance Publique-Hôpitaux de Marseille (AP-HM), Microbes, Evolution, Phylogénie et Infection (MEPHI), 13385 Marseille, France; gaye.mapenda@gmail.com (M.G.); amanzougaghene_nadia@yahoo.fr (N.A.); younes.laidoudi@yahoo.com (Y.L.); eaniang1@gmail.com (E.H.A.N.); didier.raoult@gmail.com (D.R.); 2 Institut Hospitalo-Universitaire Méditerranée-Infection, 13385 Marseille, France; maureen.laroche972@gmail.com (M.L.); jmberenger62@gmail.com (J.-M.B.); florence.fenollar@univ-amu.fr (F.F.); 3 Laboratoire d’Ecologie Vectorielle et Parasitaire, Faculté des Sciences et Techniques, Université Cheikh Anta Diop (UCAD) de Dakar, Bp 5005 Dakar-Fann, Senegal; 4 Biomedical Research Center, Institute of Virology, Slovak Academy of Sciences, Dúbravská Cesta 9, 845 05 Bratislava, Slovakia; zuzana.sekeyova@savba.sk; 5 Aix Marseille University, Institut de Recherche pour le Développement (IRD), Assistance Publique-Hôpitaux de Marseille (AP-HM), Service de Santé des Armées (SSA), Vecteurs-Infections Tropicales et Méditerranéennes (VITROME), 13385 Marseille, France; 6 Institute of Zoology, Slovak Academy of Sciences, Dúbravská Cesta 9, 845 06 Bratislava, Slovakia; maria.kazimirova@savba.sk

**Keywords:** parasitoid wasps, ticks, Western Africa, Russia

## Abstract

Some parasitoids of the genus *Ixodiphagus* (Hymenoptera, Chalcidoidea: Encyrtidae) are well-known natural enemies of ticks. In this study, we investigate the occurrence of parasitoid wasps in adult hard ticks from Western Africa (Côte d’Ivoire and Senegal) and Far Eastern Europe (Russia) using molecular methods. The morphological identification allowed the classification of 785 collected specimens of six species of ticks: *Rhipicephalus* (*Boophilus*) *microplus* (41%), *Ixodes persulcatus* (33%), *Dermacentor silvarum* (11%), *Haemaphysalis concinna* (7%), *Amblyomma variegatum* (5%), and *Haemaphysalis japonica* (3%). The newly developed MALDI-TOF MS protocol identified tick species in spite of their different storage (dried or in 70% ethanol) conditions for a long period. Molecular screening of ticks by a new standard PCR system developed in this study revealed the presence of parasitoid wasp DNA in 3% (28/785) of analyzed ticks. *Ixodiphagus hookeri* was detected in 86% (24/28) of infested ticks, including 13 *I. persulcatus*, 9 *R (B) microplus,* and one *H. concinna* and *D. silvarum.* While an unidentified parasitoid wasp species from the subfamily Aphidiinae and Braconidae family was detected in the remaining 14% (4/28) infested ticks. These infested ticks were identified as *I. persulcatus*. Our findings highlight the need for further studies to clarify the species diversity of parasitoid infesting ticks by combining molecular and morphological features. The novel molecular and MALDI-TOF MS protocols could be effective tools for the surveillance and characterization of these potential bio-control agents of ticks.

## 1. Introduction

Ticks (Ixodida) are obligate blood-sucking ectoparasites of terrestrial vertebrates almost all over the world [1]. About 10% of the 900 currently identified tick species are known to transmit different types of pathogens, such as viruses (e.g., tick-borne encephalitis virus, Crimean–Congo hemorrhagic fever virus), prokaryotes (*Rickettsia* spp., *Borrelia* spp., *Anaplasma* spp., *Ehrlichia* spp.), and eukaryotes (*Babesia* spp., *Theileria* spp.) that infect humans, domestic, and wild animals [2].

In general, ticks involved in the spread of human pathogens often feed on small mammals and birds during their immature stages, while adult ticks tend to feed on bigger herbivores and carnivores [3]. As a result, because of the pathogens they transmit and the enormous economic losses they cause, the epidemiological importance of ticks is increasing dramatically worldwide [4]. Recently, the recognized number of human diseases caused by microorganisms transmitted by ticks has increased [5,6].

Three families of ticks are known: Argasidae (soft ticks), Nuttalliellidae, and Ixodidae (hard ticks) [7,8]. This stresses the need to develop targeted methods and diversify management programs for the control of these harmful vectors.

For the prevention of pathogens transmitted by arthropods, vector control is necessary. Several tick-specific pesticides (acaricides), such as organophosphates, carbamates, and pyrethroids are used to control them [9]. However, these products became less effective due to the development of resistance in targeted tick populations [9]. In addition, some of them are highly toxic to humans [9] and many of them were banned. For this reason, biological control methods are increasingly been considered as a viable alternative to the use of pesticides, and as having a very low impact on the environment [10]. Potential biological control agents include entomopathogenic fungi, nematodes, and parasitoid wasps, which represent the most promising candidates [10].

Hymenopteran parasitoids, also called parasitoid wasps, belong to the order Hymenoptera containing two suborders (Symphyta and Apocrita), classified in 27 superfamilies (9 in Symphyta and 18 in Apocrita), 132 families with about 8423 existing genera [11]. The order Hymenoptera is one of the most diverse groups of insects with 153,088 currently existing known species, in addition to 2429 extinct species [11]. Most of the known parasitoids belong to the group of terebrants (Apocrita), comprising the superfamilies Chalcidoidea (23 families), Cynipoidea (8 families), and Ichneumonoidea (3 families) [11].

Because of their relatively high host specificity, hymenopteran parasitoids have long been recognized as important biological control agents of pests in agriculture [12]. However, the biology and ecology of these parasitoids remain largely unknown [12]. In particular, females of parasitoid wasps use their ovipositor, a spawning organ at the end of their abdomen, to lay their eggs on or into their hosts, where hatched larvae will feed-on [13]. The host eventually dies due to parasitism, although there appear to be examples where the host can survive and continue to reproduce [14,15,16].

Ticks have few natural enemies and are generally parasitized by chalcid wasps belonging to the Encyrtidae family, which have been described in many tick species worldwide [14]. Since the beginning of the 20th century, wasps of the genus *Ixodiphagus* (Chalcidoidea: Encyrtidae) are known as the only tick parasitoid. Currently, eight species of the genus *Ixodiphagus* are identified: *Ixodiphagus texanus* (Howard, 1907), *Ixodiphagus hookeri* (Howard, 1908), *Ixodiphagus mysorensis* (Mani, 1941), *Ixodiphagus hirtus* (Nikolskava, 1950), *Ixodiphagus theilerae* (Fielder, 1953), *Ixodiphagus biroi* (Erdos, 1956), *Ixodiphagus sagarensis* (Geevarghese, 1977), and *Ixodiphagus taiaroaensis* (Heath and Cane, 2010) [17,18].

These wasps, in particular I. hookeri, have been found in several species of ticks belonging to the genera Ornithodoros, Amblyomma, Dermacentor, Hyalomma, Haemaphysalis, Ixodes, and Rhipicephalus [16,19,20,21]. In contrast, the efficacy of Ixodiphagus wasps in tick control is still debated by researchers, as their biology has not been fully clarified [10]. The largest study to date has demonstrated the ability of these parasitoids to reduce the number of Amblyomma variegatum (Fabricius, 1794) ticks in a Kenyan cattle population [20]. Recently, similar studies have demonstrated the potential role of I. hookeri in the circulation of certain bacteria, such as Arsenophonus nasoniae (Gherna et al., 1991), rickettsiae (Rickettsia helvetica (Beati et al., 1993) and Rickettsia monacensis (Simser et al., 2019)), and Wolbachia (Hertig 1936) bacteria in ticks including Ixodes ricinus (Linnaeus, 1758) [22,23,24]. Unfortunately, there is a critical lack of data on the existence and/or prevalence of parasitoid wasps in ticks in Africa and Eurasia. In this context and to extend our knowledge on wasps parasitizing ticks, we present in this article molecular data of parasitoids wasps of hard ticks from Western Africa and the Russian Far East.

## 2. Materials and Methods

### 2.1. Study Area and Tick Collection

**Figure 1:**
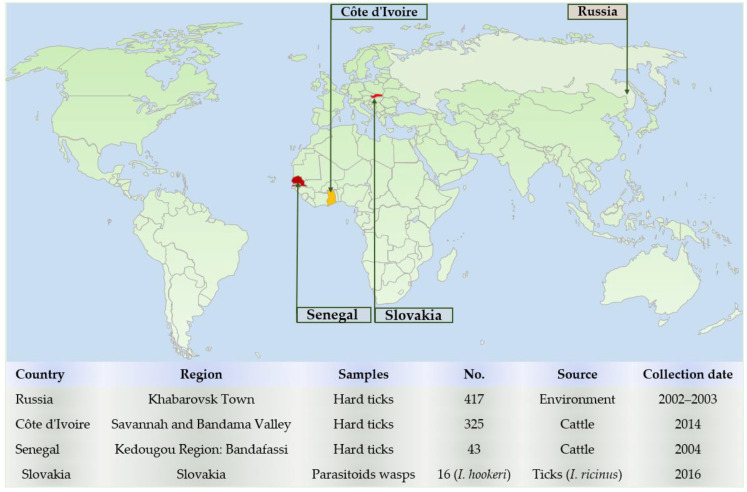
Map showing the type, origin, period, and number of samples collected in Africa and the Russian Far East.

A total of 785 hard ticks were collected from two regions of two continents (Western Africa and Russian Far East) (Figure 1). In Western Africa, ticks were collected from two countries, Côte d’Ivoire and Senegal. In Côte d’Ivoire, ticks were collected manually from cattle in the Savannah and Bandama Valley, over a period ranging from 30 October to 8 November 2014. In Senegal, ticks were collected manually from cattle in the village Bandafassi in the region of Kedougou. The collection was performed in 17 August 2004. Ticks were stored in 70% ethanol. Far Eastern questing ticks were collected in Russia near the city of Khabarovsk. The collection was performed in the Khekhtsir forest using the flag technique in May 2002 and 2003. The ticks collected were kept dried. In addition, DNA of 16 adult *Ixodiphagus hookeri* (parasitoids wasps) obtained from *Ixodes ricinus* nymphs collected in Slovakia [22] were used as positive controls in this study.

### 2.2. Morphological Identification

All tick species were identified morphologically using taxonomic keys [25], then transported and stored at the laboratory of the IHU-Mediterranean infection, Marseille (France) until further investigations.

### 2.3 Dissection and Preparation of Samples

The dissection was performed using a sterile surgical blade. Four legs on one side of each tick specimen were cut, then used for analysis by Matrix Assisted Laser Desorption Ionization—Time of Flight (MALDI-TOF, Bruker Daltonics, Bremen, Germany). Afterward, a longitudinal section was performed to obtain two equal parts of the ticks. One part was used for molecular analyzes and the second one was kept in a sterile tube and stored at −20 °C for further analysis.

### 2.4. Identification of Ticks by MALDI-TOF MS Analysis

#### 2.4.1. Sample Preparation

To gain further improvement on protein extraction for MALDI-TOF analysis, the following protocol was developed: The tick legs from the dry-stored specimens were firstly rehydrated by using adding 40 μL of HPLC water to each of the tubes containing the tick samples for 24 h prior to the homogenization. Subsequently, a pinch of glass powder (Sigma, Lyon, France) was added to each sample, plus 40 μL of a 70% (*v*/*v*) mixture of formic acid and 50% (*v*/*v*) acetonitrile (Fluka, Buchs, Switzerland). Finally, the mixture was crushed using Tissue Lyser (QIAGEN, Hilden, Germany) and centrifuged at 2000× *g* for 30 s. The supernatant was recovered and used for MALDI-TOF MS (Bruker Daltonics, Bremen, Germany) identification [26].

#### 2.4.2. Setting Up Samples on the Target Plate

The previous mixtures containing the crushed legs were centrifuged at 2000× *g* for 30 s. Four replicates of 1 μL of the supernatant from each sample was carefully placed on a MALDI-TOF target plate and were then dried at room temperature, as described elsewhere [26]. One microliter of CHCA matrix solution composed of saturated α-cyano-4-hydroxycynnamic acid (Sigma, Lyon France), 50% of acetonitrile (*v*/*v*), 2.5% of acid trifluoroacetic acid (*v*/*v*) (Aldrich, Dorset, UK) and HPLC grade water was deposited on each spot of the target plate. The target plate was then dried at room temperature and was then introduced directly into the MALDI-TOF Microflex LT mass spectrometry device (Bruker Daltonics, Bremen, Germany) for analysis [27].

#### 2.4.3. MALDI-TOF Parameters (MS)

In this study, the protein mass profiles for each tick sample were obtained using a MALDI-TOF Microflex LT mass spectrometer (Bruker Daltonics) with the Flex Control software (Bruker Daltonics). Spectra record was performed in a linear positive ion mode with an acceleration voltage of 20 kV within a mass range of 2–20 kDa. A total of 240 laser shots were used to generate each spectrum from six regions of the same spot. The analysis of the spectrum profiles obtained was made by Flex analysis software v.3.3. Spectrum profiles were subsequently exported to another ClinProTools v.2.2 and MALDI-Biotyper v.3.0 software (Bruker Daltonics) [28,29].

#### 2.4.4. Database Implementation and Blind Test

Four ticks species (*Dermacentor silvarum, Haemaphysalis concinna, Haemaphysalis japonica,* and *Ixodes persulcatus*) from the present study did not have spectral profiles in MALDI-TOF (MS) database of arthropods in IHU laboratory. The reference spectra for each species were created and added to the database. The reference spectra were assessed against the MALDI-TOF (MS) database reference spectra database using the blind test [30]. The reliability of species identification was estimated using the log score values (LSVs) obtained from the MALDI-Biotyper software v.3.3, which ranged from 0 to 3. These LSVs correspond to the degree of similarity between the MS reference spectra database and those submitted by blind tests.

### 2.5. Molecular Analysis

#### 2.5.1. DNA Extraction

Genomic DNA was extracted individually from the half-part of each tick and from the *I. hookeri* specimens. In order to minimize PCR inhibitors from tick samples, we followed the extraction protocol described by Halos et al., 2004 [31]. Briefly, a bead-based physical disruption of the tick body within the Tissue-Lyser apparatus (Qiagen, Hilden, Germany), and 24 h of enzymatic digestion at 56 °C using buffer G2 supplemented with 25% of proteinase K were performed prior DNA extraction. Meanwhile, specimen *I. hookeri* subjected to enzymatic lysis with proteinase K followed by incubation at 56 °C overnight prior to DNA extraction. The extraction was performed using the QIAamp Tissue Extraction Kit (Qiagen, Courtaboeuf, France), in the QiagenEZ1 automated system, following the manufacturer’s instructions. DNA was eluted in 100 μL and stored at −20 °C until further use.

#### 2.5.2. Standard PCR and Sequencing

Primers (28S-hym-F:5′-AGACCGATAGCGAACAAGTA-3′; 28S-hym-R: 5′-GGTCCTGAAAGTACCCAAA-3′), targeting partial 28S ribosomal RNA (28S rRNA) gene (560 bps), were designed by alignment of sequences from a wide range of closely related hymenopteran parasitoids available from GenBank database, using Primer3 software, version 4.0 (http://frodo.wi.mit.edu/primer3/) following the general rules described elsewhere [32]. Subsequently, the combinations forward-reverse was checked within the DNA databases of metazoans (taxid:33,208), vertebrates (taxid:7742), bacteria (taxid:2), Canidae (taxid:9608), Felidae (taxid:9682), and humans (taxid:9605) using primer-BLAST [33]. Meanwhile, the in vitro validation was performed by challenging the newly designed primers against DNA collection from a panel of arthropods and bacterial DNAs (Table S1).

Once validated, the PCR was used from the screening for potential hymenopteran parasitoid DNA from all tick samples. Standard PCR protocol was performed in a Thermal Cycler Peltier PTC200 cycler thermal model (MJ Research Inc., Watertown, MA, USA). Each reaction was conducted in a final volume of 50 μL, containing 5 μL of DNA of each sample, 25 HotstarTaq—AmpliTaq Gold (Life Technologies, Carlsbad, CA, USA), 1.5 μL of primers (Forward and Reverse) and 17 μL water DNAse/RNAse free, using the following thermal cycling conditions: an initial denaturation step at 95 °C for 15 min, followed by 40 denaturation cycles at 95 °C for 30 s, step hybridization at a temperature of 59 °C for 30 s, and an elongation at 72 °C for 30 s. The DNA of *I. hookeri* was used as a positive control and master mixture as a negative control for each experiment. All PCR products were resolved in 0.5x GelRed stained (Biotium, CA, USA) agarose gels (2%), purified using NucleoFast 96 PCR plates (Macherey-Nagel EURL, Hoerdt, France) and sequenced using the Big Dye Terminator Cycle sequencing kit (Perkin Elmer Applied Biosystems, Foster City, CA, USA) with an automated ABI sequencer (Applied Biosystems, Foster City, CA, USA).

#### 2.5.3. Phylogenetic Analysis

DNA sequences were assembled using ChromasPro software (ChromasPro 1.7, Technelysium Pty Ltd., Tewantin, Australia). Indels, stop codons, and ambiguities were visually cheeked and resolved. Species resolution was firstly performed using BLASTn analysis within the Basic Local Alignment Search Tool (BLAST) [34]. The 28S rRNA sequences were aligned against the homologous GenBank sequences from the closely related species. The alignment was performed using MAFFT [35]. Best fit model and maximum likelihood phylogeny were performed on MEGA 6 [36]. Phylogram was edited using iTOL v4 software [37]. Additionally, the interspecific nucleotide pairwise distance (INPD) was evaluated to estimate the genetic divergence between species we amplified herein and hymenopteran parasitoids from GenBank database using MEGA 6 [36].

## 3. Results

### 3.1. Tick Collection and Morphological Identification

**Figure 2:**
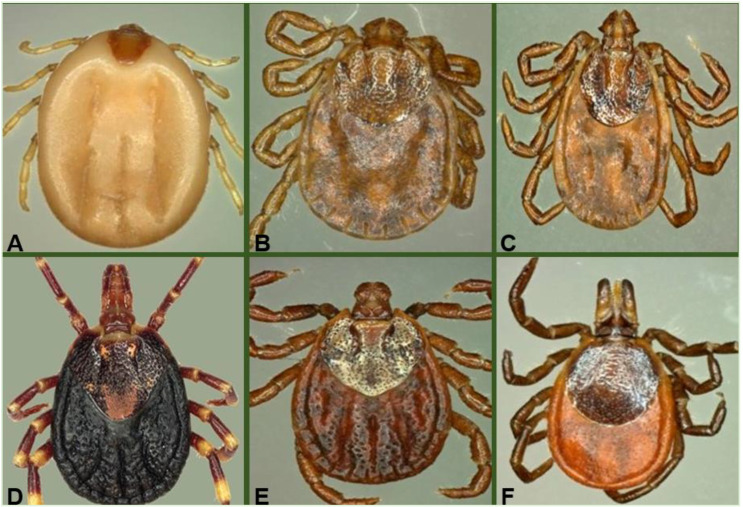
Pictures of hard tick species collected and studied from Western Africa and the Far East. (**A**) *Rhipicephalus (Boophilus) microplus*, (**B**) *Haemaphysalis japonica*, (**C**) *Haemaphysalis concinna*, (**D**) *Amblyomma variegatum*, (**E**) *Dermacentor silvarum,* and (**F**) *Ixodes persulcatus*.

A total of 785 adult hard ticks were collected in Western Africa (Senegal and Côte d’Ivoire; *n* = 368) and the Russian Far East (*n* = 417). Morphological identification revealed the presence of six tick species, belonging to five genera (Figure 2). In Western Africa, the species identified were: *A. variegatum* (43 including 5 males and 38 females) from Senegal and *Rhipicephalus (Boophilus) microplus* (325 including 65 males and 260 females) collected from Côte d’Ivoire. Ticks from the Russian Far East belonged to the following species: *I. persulcatus* (256 of which 132 were males and 124 females), *D. silvarum* (83: 48 males and 35 females), *H. concinna* (54: 29 males and 25 females), and *H. japonica* (24: 8 males and 16 females) (Table 1).

### 3.2. Analysis of MALDI-TOF MS Spectra

**Figure 3:**
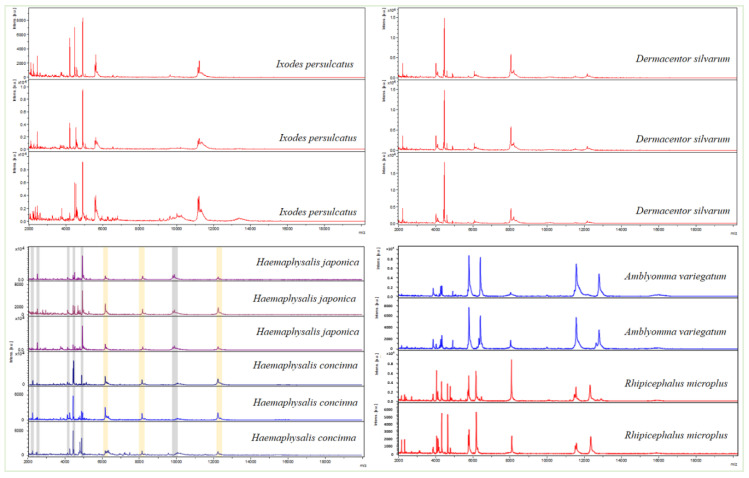
MS spectra profiles of female and male tick species using the new “rehydration” protocol. Species and genus-specific spectra are respectively indicated in grey and yellow colons for *Haemaphysalis* ticks.

A total of 84 tick specimens (7 males and 7 females per species) representing the six different species were analyzed by MALDI-TOF (MS). Visual comparison of spectrum profiles using the gel view tool and superposition of spectrum profiles in each state using ClinProTools software (Bruker Daltonics, Bremen, Germany) revealed a clear difference in spectral profiles of the different tick species. Analysis of the spectral profiles using this new protocol (rehydration) exhibited good reproducibility quality of the spectra of the six tick species. MS spectra provided correct identification at the species level with the ability to distinguish between species from the same genus. Spectra from *H. concinna* and *H. japonica* shared at least three conserved peaks corresponding to the genus; while species were differentiated by six peaks wherein the intensity was significantly different between *Haemaphysalis* species for each peak (Figure 3).

All spectra of the 84 specimens, including 14 *D. silvarum*, 14 *I. persulcatus*, 14 *A. variegatum*, 14 *R. (B.) microplus*, 14 *H. concinna,* and 14 *H. japonica*, were screened against the arthropod database (DB) presenting tick reference spectra. Among the six species, only two spectra (*A. variegatum* and *R. (B.) microplus*) existed in the DB. The blind test against DB revealed an identification of only two species *A. variegatum* and *R. (B.) microplus*. Then, the spectra of the other morphologically identified species, namely *D. silvarum*, *I. persulcatus*, *H. concinna,* and *H. japonica*, were added to this database.

### 3.3. Molecular Detection of Parasitoid Wasps and Phylogenetic Analysis

Both in-silico and in-vitro validations revealed that the newly designed primers were specific for the target species. The expected DNA amplicons were successfully obtained from *I. hookeri specimen*, while no DNA amplification was obtained from the panel of negative controls summarized in Supplementary Table S1.

Among the 785 ticks screened for the presence of parasitoid wasps’ DNA, 3% (28/785) were positive. This include: *I. persulcatus* (*n* = 17), *R. (B.) microplus* (*n* = 9), *D. silvarum* (*n* = 1), and one *H. concinna* sample. Meanwhile, DNA amplicons were obtained from *A. variegatum* nor *H. japonica* specimens. All positive ticks as well as the positive controls (*I. hookeri*) yielded reliable DNA sequences. The partial DNA sequences of the 28S rRNA gene obtained from this study were deposited in the GenBank under accession numbers from MN956813 to MN956823.

Blast analysis showed that, among the 28 DNA sequences, 24 (85.7%) of them had an identity ranged from 99.6 to 100% with the positive control (*I. hookeri,* GenBank accession number MH077537). The remaining four sequences (4/28), amplified from *I. persulcatus* from Russia, were all identical to each other and showed an identity ranged from 97.2% to 97.7% and a query cover from 95.3% to 97.6% with Aphidiinae species (KP983290, FJ396357, and FJ396381). More specifically, these 24 sequences harbored nine different genotypes (Table 2).

**Figure 4:**
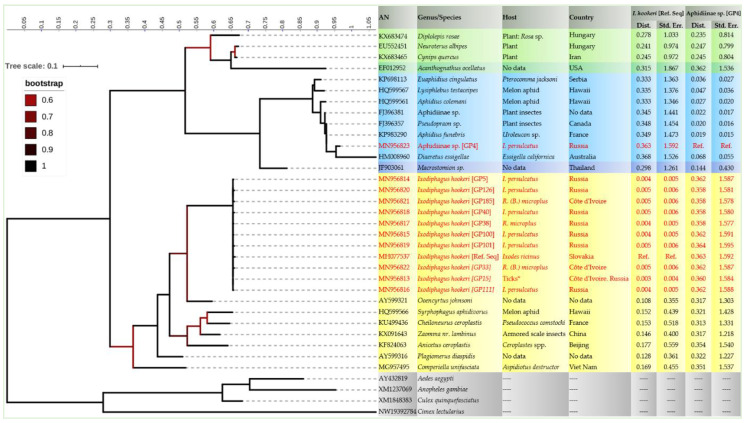
Phylogenetic tree showing the position of *I. hookeri* and Aphidiinae sp. GP4 genotypes obtained in the present study (indicated in red) among the representative members of hymenopteran parasitoids families. The tree was inferred using the Maximum Likelihood method based on 1000 bootstraps and the Kimura 2-parameter model [38]. The analysis involved 35 partial 28S DNA sequences. There were a total of 646 positions in the final dataset. Outgroup taxons Culicidae (AY432819, XM1237069 and XM1848383) and Cimicidae (NW19392784) are drawn at root. The highest log likelihood was −7750.7467. The axis showed the global distance observed throughout the trees. Branches are color-coded according to the bootstrap’s percent. The identity of each taxa is color-coded according to the family/subfamily (pistachio green: Cynipidae/Cynipinae; green: Formicidae/Myrmicinae; light blue: Braconidae/Aphidiinae; yellow: Encyrtidae/Encyrtinae; grey: outgroup taxons). GenBank accession numbers, species, hosts and geographical origin when available are indicated at each node. The number of base substitutions and the Standard error estimate(s) per site from between the reference sequences of *I. hookeri* and Aphidiinae sp. genotype GP4 and hymenopteran parasitoids from GenBank are shown. Analyses were conducted using the Maximum Composite Likelihood model [39]. All ambiguous positions were removed for each sequence pair (pairwise deletion option).

Phylogenetic analysis (Figure 4) showed that all *I. hookeri* genotypes clustered with the reference sequence of *I. hookeri* (MH077537) and where monophyletic with the hymenopteran parasitoids of plants-associated insects belonging to the family Encyrtidae and the subfamily Encyrtinae. Accordingly, the INPD between the reference sequence of *I. hookeri* and those herein amplified from ticks ranged from 0.003 with the most prevalent genotype (GP15, GenBank accession number: MN956813) to 0.004–0.005 with the other genotypes. Among the other member of Encyrtinae subfamily, *I. hookeri* showed a distance ranged from 0.108 with *Ooencyrtus johnsoni* (GenBank accession number: AY599321) to 0.177 with *Anicetus ceroplastis* (GenBank accession number: KF824063). However, the genotype amplified from the Russian *I. persulcatus* clustered monophyletically with the other members of the family Braconidae and the subfamily Aphidiinae, hymenopteran parasitoids of plants-associated insects. The INPD between this potential new *species*, provisionally referred here as “Aphidiinae sp. GP4” and the other Aphidiinae species ranged from 0.019 with *Aphidius funebris* (GenBank accession number: KP983290) to 0.068 with *Diaeretus essigellae* (GenBank accession number: HM008960).

## 4. Discussion

A new standard PCR system targeting the partial sequence of the 28S rRNA gene was developed for the detection of parasitoid Hymenoptera and could be used for routine screening of wasps in ticks as well as in other hosts. This system seems to be sensitive and specific. The amplified 28S rRNA gene portion makes it possible to distinguish between the different species of hymenopterans.

Screening for the presence of parasitoid wasp’s DNA revealed a positivity of 3.6% (28/785). Such a low prevalence of parasitoid wasp infestation can be explained by the fact that only adult tick specimens were analyzed. Indeed, infected ticks die because of parasitism, but, eventually, some can survive and transform into adult [14,16,18], so the infection level is usually much more important at immature stages compared to adults [40]. A recent study on *I. ricinus* showed that the *I. hookeri* wasp infestation was higher in nymphs (infestation rate 7.2% to 14.6%) than in adults (0.6%; 3/481) [40]. In the site in Slovakia, from where the *I. hookeri* positive controls originated, the infestation rate of *I. ricinus* nymphs during 2015–2017 ranged between 4.1% and 23.5% (M. Kazimírová, unpublished). Here, we show that parasitoid wasps’ infestation can occur in adult ticks, and with a higher prevalence than previously reported. If wasps are detected in adult ticks, the development of parasitoid wasp larvae (e.g., *I. hookeri*) is unlikely to occur successfully [40].

In this study, we report for the first time the presence of *I. hookeri* in ticks from Western Africa (Côte d’Ivoire) in *R. (B.) microplus*) and in Russian Far East (in *I. persulcatus*, *D. silvarum* and *H. concinna*). It has been shown that wasps of the genus *Ixodiphagus* have a wide host range [18]. Recent studies have reported the ability of *I. hookeri* to infest several tick species belonging to the genera *Ornithodoros*, *Amblyomma*, *Dermacentor*, *Hyalomma*, *Haemaphysalis*, *Ixodes,* and *Rhipicephalus* [18,19,21]. These results confirm the global distribution of *I. hookeri* reported by other studies, particularly in Africa [20], America [19], and Europe [22,23,24].

Parasitoid sequences obtained from four (4/28) *I. persulcatus* ticks were 100% identical to each other. These sequences probably represent a not yet described species within the Aphidiinae subfamily, Braconidae family, as low identity with only 97.2–97.7% and its INPD > 0.36 as well as its position on phylogenetic tree suggest it. *All closest matches for these sequences belong to* parasitoid wasps of the Braconidae family (Hymenoptera: Braconidae). Parasitoids from this family were not previously known to parasitize hard ticks. To date, only parasitoid wasps of the genus *Ixodiphagus* (Hymenoptera: Encyrtidae) have been reported as tick parasites [18].

The genetic variant identified in this study probably belongs to a new wasp species of the family Braconidae. Amplification of other genes (such as mitochondrial genes) followed by phylogenetic analysis, may be necessary to better describe this new genetic variant. Species of the *Aphidius* genus are known to parasitize aphids, but no species of this genus have been reported so far in ticks [41]. Therefore, the Braconidae parasitoid wasp found in *I. persulcatus* should be necessarily identified by prospective entomological studies. It is interesting to note that the Russian Far East is the only region from which *I. hirtus*, another encyrtid wasp parasitizing *I. persulcatus* is known. However, even taking into consideration morphological differences between *I. hirtus* and other species of *Ixodiphagus* genus, the sequence obtained during the present study cannot be attributed to *I. hirtus*, because 28S-based genetic identities between Encyrtidae (for example *Ixodiphagus* sp.) and Braconidae are never higher than 92%. Therefore, hymenopteran sequences identified in *I. persulcatus* ticks certainly do not belong to Encyrtidae.

The identification of ticks collected in Western Africa and the Russian Far East by MALDI-TOF MS using a new rehydration protocol, has allowed the incrementation of our arthropods database with the new spectra of four hard tick species, namely *I. persulcatus*, *D. silvarum*, *H. concinna*, and *H. japonica*. Moreover, the new protocol proposed in this study based on the rehydration of alcohol-stored specimens, generated reproducible spectra even for old tick samples stored dry or in 70% ethanol. Indeed, even if kept dry for at least 15 years, this protocol could give profiles of mass spectrometry spectra (MS) specific to different tick species. Therefore, new protocol used during this study could be standardized for the routine identification of arthropod specimens kept for a long time making them difficult to identify by classical morphological identification keys. These results confirm findings of recent studies indicating that MALDI-TOF (MS) could be used as a rapid, reliable, inexpensive, and effective tool for the identification of tick species [28,30] as well as species of other arthropod vectors [42]. However, the conservation status of arthropod specimens may influence the quality and reproducibility of spectra, and an earlier study showed that the use of fresh or frozen specimens could improve the reproducibility and quality of spectra [26]. The MALDI-TOF (MS) has revolutionized clinical microbiology by its effectiveness in the routine identification of microorganisms [43]. Now, it appears to be effective for the rapid identification of arthropods of medical interest requiring no expertise in arthropod identification [26,30,44]. Currently, this innovative tool is increasingly being used, as it presents simple and fast data analysis compared to morphological and molecular methods [45].

## 5. Conclusions

In this study, we phylogenetically describe hymenopteran parasitoids belonging to two distinct families: *I. hookeri* (Encyrtidae, Encyrtinae) in adult hard ticks from Western Africa (*R. (B.) microplus*) and the Russian Far East (*I. persulcatus*, *D. silvarum,* and *H. concinna*) and an unidentified Aphidiinae sp. (Braconidae, Aphidiinae) in Russian *I. persulcatus.* In addition to the wide distribution of these parasitoids, this study reports an Aphidiinae sp. apparently parasitizing *I. persulcatus* for the first time. Phylogenetic analysis highlights the necessity of completing the classification of parasitoids infesting ticks by combining morphological and molecular-based identification for an integrative taxonomical perspective.

## Figures and Tables

**Table 1:** Ticks species, location, and parasitoid wasps’ infection.

Country/Region	Geographic Coordinates	Tick Species	Number (Male/Female)	Parasitoid Wasps (%)
Russia/Khabarovsk	48°28′57″ N 135°05′01″ E	*Ixodes persulcatus*	256 (132/124)	17 (7%)
		*Dermacentor silvarum*	83 (48/35)	1 (1%)
		*Haemaphysalis concinna*	54 (29/25)	1 (2%)
		*Haemaphysalis japonica*	24 (8/16)	0
Côte d’Ivoire/Bandama Valley and Savannah	8°8′ N 5°6′ W	*Rhipicephalus (Boophilus) microplus*	325 (65/260)	9 (3%)
Senegal/Bandafassi	12°32′ N, 12°19′ W	*Amblyomma variegatum*	43 (5/38)	0
Total			785 (287/498)	28 (3%)

**Table 2:** Sequences of parasitoid wasps amplified in this study and deposited in GenBank.

Sequences Type	Genotype	Tick Species (no.)	Collection Site	Total	Ascension Number
*I. hookeri*	GP15	*R. (B.) microplus* (6) *I. persulcatus* (7) *D. silvarum* (1) *H. concinna* (1)	Côte d’Ivoire, Russia	15	MN956813
*I. hookeri*	GP5	*I. persulcatus* (1)	Russia	1	MN956814
*I. hookeri*	GP100	*I. persulcatus* (1)	Russia	1	MN956815
*I. hookeri*	GP111	*I. persulcatus* (1)	Russia	1	MN956816
*I. hookeri*	GP38	*R. microplus* (1)	Russia	1	MN956817
*I. hookeri*	GP40	*I. persulcatus* (1)	Russia	1	MN956818
*I. hookeri*	GP101	*I. persulcatus* (1)	Russia	1	MN956819
*I. hookeri*	GP126	*I. persulcatus* (1)	Russia	1	MN956820
*I. hookeri*	GP185	*R. (B.) microplus* (1)	Côte d’Ivoire	1	MN956821
*I. hookeri*	GP33	*R. (B.) microplus* (1)	Côte d’Ivoire	1	MN956822
Aphidiinae sp.	GP4	*I. persulcatus* (4)	Russia	4	MN956823
**Total**		4		28	

## Supplementary Materials

The following are available online at https://www.mdpi.com/2076-2607/8/12/1992/s1, Table S1: Analytical specificities: a panel of arthropods and bacterial species tested with Hymenoptera-specific 28S rRNA based standard PCR assays.
