# Supplementary material for: Hymenopteran Parasitoids of Hard Ticks in Western Africa and the Russian Far East
Source: Microorganisms. 2020 Dec 14;8(12):1992. doi: 10.3390/microorganisms8121992 (PMC7765078; doi:10.3390/microorganisms8121992)
Supplement: Supplementary file 1 [file microorganisms-08-01992-s001.zip › Table S1.docx]

| **Panel** | **Standard PCRs results** |
| --- | --- |
| *Ixodiphagus hookeri* | Positive |
| *Blatella germanica* | Negative |
| *Plasmodium falciparum* | Negative |
| *Periplaneta americana* | Negative |
| *Rickettsia typhi* | Negative |
| *Rickettsia africae* | Negative |
| *Bartonella quintana* | Negative |
| *Borrelia recurrentis* | Negative |
| *Tropheryma whipplei* | Negative |
| *Staphylococcus aureus* | Negative |

**Table S1. Analytical specificities: a panel of arthropods and bacterial species tested with Hymenoptera-specific 28S-based standard PCR assays.**
